# The effectiveness of exercise and/or nutritional interventions to improve the quality of life of women with breast cancer receiving radiation therapy: a scoping review

**DOI:** 10.1007/s00520-024-08933-1

**Published:** 2024-10-23

**Authors:** Laura Feighan, Lesley MacDonald-Wicks, Robin Callister, Yolanda Surjan

**Affiliations:** 1 Present Address: Global Centre for Research and Training in Radiation Oncology, School of Health Sciences, College of Health, Medicine and Wellbeing, The University of Newcastle, Callaghan, Newcastle, NSW Australia; 2 School of Health Sciences, College of Health, Medicine and Wellbeing, The University of Newcastle, Callaghan, Newcastle, NSW Australia; 3 School of Biomedical Sciences and Pharmacy, College of Health, Medicine and Wellbeing, The University of Newcastle, Callaghan, Newcastle, NSW Australia

**Keywords:** Breast cancer, Radiation therapy, Diet, Nutrition, Exercise, Quality of life, Survivorship

## Abstract

Currently, in Australia, 1 in 8 women are diagnosed with breast cancer. A common adjuvant treatment for breast cancer is radiation therapy (RT). The amalgamation of side effects caused by RT treatment can ultimately affect a patient’s quality of life (QoL). With increasing breast cancer survival, a greater focus on the non-lethal consequences of this disease and its treatment is warranted. Exercise and nutrition have proven beneficial in promoting and supporting overall health and managing chronic diseases. Furthermore, exercise has demonstrated improvement and sustainment to QoL. The focus of this scoping literature review was to determine the scale of evidence regarding the effectiveness of exercise and/or nutritional interventions for women with breast cancer receiving radiation therapy. An online search of five databases was conducted to identify studies published between 2000 and 2023. The 58 studies included in the scoping review comprised 46 interventions and 4615 women with breast cancer who received radiation therapy participated. Most studies (90%; *n* = 52) were ‘exercise only’ based, 3% (*n* = 2) were ‘nutrition only’, and the remaining 7% (*n* = 4) of studies were combined exercise and nutrition interventions. The findings from this review highlight most studies are dedicated to investigating exercise. Further research is required to fully understand the potential benefits of these interventions and their synergistic impact on the quality of life of women with breast cancer receiving radiation therapy.

**Supplementary Information:**

The online version contains supplementary material available at 10.1007/s00520-024-08933-1.

## Introduction

Breast cancer is an insidious disease that affects 1 in 8 Australian women [1]. Radiation therapy (RT) treatment for breast cancer is widely utilised as an adjuvant treatment, generally occurring post-surgery and chemotherapy. RT is employed to maintain local control and prevent recurrence. The degree of treatment is dependent on the extent of the disease, including treatment to the whole breast following breast-conserving surgery and chest wall treatment post-mastectomy, and may incorporate treatment to diseased lymph nodes [2].

While the continual advancements of RT provide precision accuracy to target volumes, it is difficult to completely spare surrounding healthy tissues and organs in the process [3]. Consequently, RT can cause side effects, which are dependent on the site of the body being treated, the radiation prescription, and the demographic characteristics of the patient (such as age and comorbidities). For breast cancer, common side effects include skin reactions, fatigue, and lymphoedema [4]. The amalgamation of such RT-associated side effects can adversely affect a patient’s quality of life (QoL) [5].

QoL is a multidimensional concept that considers all characteristics impacting a person’s life and well-being. The World Health Organisation defines QoL as an *‘individuals’ perception of their position in life in the context of culture and value systems in which they live and in relation to their goals, expectations, standards and concerns’* [6]. Health-related QoL includes information on the physical and mental health of individuals and the impact of health status on QoL [7]. Alongside physical side effects of RT, women with breast cancer can experience anxiety and depression, compromised sleep quality, mood disorders and impaired physical functioning, weight and body composition changes, stress, and self-esteem depletion [8].

Due to the continually improving medical and technological developments in breast cancer treatment, the current survival rate for the Australian population is rapidly improving, with the current relative survival rate being 92% [1]. With breast cancer survival rates rising, a greater focus on the non-lethal consequences of this disease and its treatment is essential. QoL is now deemed an important aspect when considering a patient’s treatment pathway. It is recognised as a critical outcome variable separate to medical or clinical outcomes and can be included in the deliberation of a patient’s prognosis [9, 10]. Furthermore, in view of the improving breast cancer survival rate, there is a growing demand to implement high-quality strategies for women in the survivorship phase. Survivorship refers to a person’s well-being from cancer diagnosis to the end of life. This includes all aspects of QoL and can encompass late toxicity and lifestyle interventions [11].

Two interventions often proposed to have positive effects on QoL in multiple contexts, including cancer, are improvements to diet and exercise quality [12, 13]. Ruegsegger et al. discuss the support physical activity and exercise provide in both physical and mental health, including the connection between inactivity and the development of chronic disease (such as coronary heart disease and hypertension) and psychological disorders (such as depression and anxiety) [14]. Moreover, an exploration of the diet quality and QoL in Australians between 55 and 65 years was undertaken by Milte et al. where it was found that adherence to a healthy diet was associated with better QoL [15].

There is currently a lack of exercise and dietary guidance available for women with breast cancer receiving RT. Insufficient information exists on how exercise and nutritional advice could enhance their treatment experience and recovery. Literature has traditionally focused on diagnoses like gastrointestinal and head and neck cancer, due to the acute RT side effects patients experience (such as nausea, vomiting, and difficulty swallowing) [16, 17]. Furthermore, there is a lack of literature specifically focusing on RT treatment, with ‘cancer treatment’ used as an umbrella term for the various treatment options patients may undertake, despite the vast differences in the side effects and impact on the patient’s lifestyle of each treatment type [18, 19].

The primary aim of this scoping review was to identify the existing literature reporting the use of exercise and/or nutritional interventions to improve an aspect of QoL for women with breast cancer receiving radiation therapy. Secondary objectives were to summarise the characteristics of these interventions and the aspects of QoL reported. Understanding the impact of exercise and nutrition on women with breast cancer receiving RT could potentially improve their QoL, health outcomes, and RT experience.

## Methods

This literature review was conducted using the PRISMA guidelines for scoping reviews (Prisma-SCR) [20].

### Search strategy

An online search of the Medline, CINAHL, Embase, Cochrane, and SCOPUS databases was conducted between 2000 and October 2023 in conjunction with a medical librarian, using predefined keywords (for the full search strategy, see Supplementary Material).

### Eligibility criteria

The Population, Intervention, Control, Outcomes and Study Design (PICOS) framework was implemented to specify inclusion criteria (see Table 1) [21].

**Table 1:** PICOS framework for inclusion/exclusion criteria

**Population**	**Included**
	1. Females
	2. 18 years +
	3. Diagnosed with breast cancer
	4. Treatment included RT
	**Excluded**
	1. Males
	2. Females under 18 years
	3. Not diagnosed with breast cancer
	4. Treatment did not include RT
	5. Interventions that are not related to exercise and/or nutrition
**Intervention**	Only literature with exercise and/or nutrition-specific interventions will be considered for inclusion. As there is a vast range of exercise and nutrition subtypes, all will be included and categorised, enabling comparison. Examples of intervention subtypes to be included: • Exercise: aerobic/cardiovascular, resistance/strength, walking, Yoga, tai chi, Qigong, Nia, dance, stretching, Pilates • Nutrition: change to nutrients, food groups, diet types, dietary patterns, diet quality The exposure group will be those who participate in an exercise and/or nutrition intervention
**Comparator**	Comparators will be those that do not participate in an exercise and/or nutrition intervention
**Outcome**	To be included in this review, studies must assess exercise and/or nutrition as intervention/s for women with breast cancer receiving RT
**Study design**	All study designs, full-text articles from the year 2000 to present, published in English, peer-reviewed journals

### Quality of life measures

As QoL is a broad term, encompassing many aspects of a person’s life, the term QoL was included in the review as a general term and included various elements of QoL (such as fatigue, depression, mood states, and physical functioning). This was to ensure any variable of the six domains of QoL were reported on, making for a thorough review of QoL impact. The six domains of QoL as per the World Health Organisation include physical, psychological, level of independence, social relationships, environment, and spiritual/religion/personal beliefs [6].

### Study selection and data extraction

The online platform Covidence (Covidence systematic review software, Veritas Health Innovation, Melbourne, Australia. www.covidence.org) was used to facilitate the screening, full-text review, and data extraction process. After duplicate articles were removed, title and abstract screening was performed by two researchers, who checked for relevance. Subsequently, the same researchers independently reviewed the full texts to identify eligible articles. Consensus of conflicts was achieved via a third researcher’s review. The study details, intervention/s, outcome measures, and outcomes were extracted by two researchers independently. For all disagreements, a consensus was achieved by a third researcher.

## Results

### Study selection

**Fig. 1:**
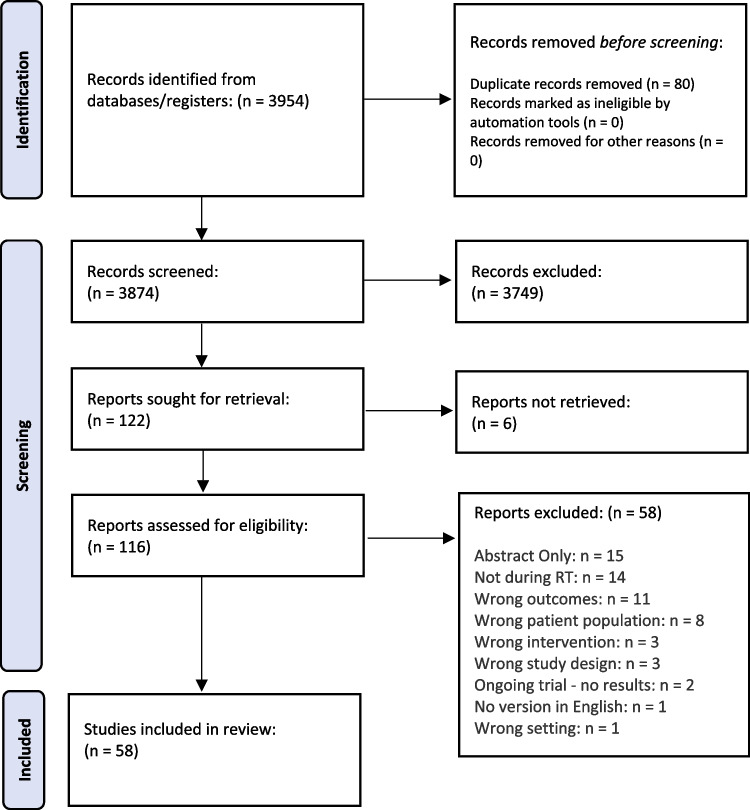
Scoping review flowchart. [20]

In total, 3954 articles were identified from the five database searches. Following the removal of 80 duplicates, 3874 titles and abstracts were screened, and 3749 articles were excluded. Reasons for exclusion included wrong cancer diagnosis, wrong stage of treatment, wrong intervention, and/or wrong outcomes. After 116 full texts were screened, 58 studies were eligible for inclusion in the scoping review. A PRISMA flowchart of the study selection process is in Fig. 1.

### Study designs

Most studies (86%; *n* = 50) (Table 2) were randomised controlled trials (RCTs) where participants were randomly assigned to either an intervention group or a control group (who received usual care). A total of 55 interventions were reported from the total 58 studies (i.e., two interventions published three studies, and another intervention published two studies). The duplicate studies were included in this review due to the authors reporting on different outcome measures from the same intervention.

**Table 2:** Details of scoping review studies

Author Year Country		Age Breast cancer stage		Number of participants		Measures		Assessment		Type of exercise		Exercise period		Nutrition focus		Nutrition consultation/education timeline		Nutrition intervention method	Outcomes
**‘Exercise-only’ studies**																			
**During active RT treatment** **only** **(period in which patient is receiving RT)**																			
Adams-Campbell et al 2023 USA [22]		18–75 years 0A–IIIA		Total: 30 Received RT: 30		HRQoL/QoL; fatigue		FACT-B; FACIT-F		Aerobic/cardiovascular (moderate intensity)		‘For the duration of RT’—weeks NR, 5 sessions per week, 15 min per session							Improvement in QoL Reduction in fatigue
Addington et al 2018 USA [23]		> 18 years 0–III		NR		Shoulder/arm ROM		Questionnaire (self-reported); Telephone interview; Yoga instructor completed a qualitative survey		Yoga		6 weeks, 2 sessions per week, 75 min per session Not clear							
Banerjee et al 2007 India [24]		30–70 years II–III		Total: 68 Received RT: 68		Anxiety; depression; stress		HADS; perceived stress scale		Yoga		6 weeks, sessions per week NR, 90 min per week							Reduction in depression; anxiety; stress
da Silva Leal et al 2016 Brazil [25]		Average: 54–55 years 0–III		Total: 35 Received RT: 35		Shoulder/arm ROM; perimetry of the upper limbs		Device (armband), Physiotherapy evaluation		Kinesiotherapy of the upper limbs		5–6 weeks, 2 sessions per week, session duration NR							Improvement in ROM
Chandwani et al 2010 USA [26]		> 18 years 0–III		Total: 71 Received RT: 71		HRQoL/QoL; fatigue; sleep quality; anxiety; depression		SF-36; BFI; PSQI; CES-D; STAI; IES; BFS;		Yoga		6 weeks, 2 sessions per week, 60 min per session							Improvement in QoL No change in depression; fatigue; sleep quality; anxiety
Chandwani et al 2014 USA [27]		> 18 years 0–III		Total: 163 Received RT: 163		HRQoL/QoL; fatigue; sleep quality; depression; physical functioning; cortisol levels		SF-36; BFI; CES-D; saliva samples		Yoga		6 weeks, 3 sessions per week, 60 min per session							Improvement in QoL; physical functioning; general health Reduction in fatigue Steeper cortisol slope in yoga group No change in sleep quality
Chen et al 2013 China [28]		> 18 years 0–III		Total: 100 Received RT: 100		HRQoL/QoL; fatigue; sleep quality; depression; cortisol rhythm		FACT-G; CES-D; BFI; PSQI; saliva samples		Qigong exercise		5–6 weeks, 5 sessions per week, 40 min per session							Improvement in QoL Reduction in fatigue; depression
Drouin et al 2005 USA [29]		20–65 years 0–III		Total: 21 Received RT: 21		Fatigue; mood state; physical functioning		R-PFS; POMS		Walking; individual exercise prescriptions		6–7 weeks, 3–5 sessions per week, 20–45 min per session							Improvement in physical functioning; mood state (depression-dejection; anger-hostility) Reduction in fatigue No change in mood state (tension-anxiety; vigour-activity; fatigue-inertia; confusion-bewilderment)
Ho et al 2016 China [30]		> 18 years 0–III		Total: 139 Received RT: 139		HRQoL/QoL; fatigue; sleep quality; anxiety; depression; stress; pain		HADS; perceived stress scale; FACT-B; BFI; Brief Pain Inventory; PSQI		Dance movement therapy		3 weeks, 2 sessions per week, 90 min per session							Improvement in QoL; sleep quality Reduction in fatigue; stress; pain No change in depression; anxiety
Hwang et al 2008 Korea [31]		30–70 years NR		Total: 37 Received RT: 37		HRQoL/QoL; fatigue; shoulder/arm ROM; pain		WHOQOL-BREF; BFI; physiotherapy evaluation		Aerobic/cardiovascular (intensity not reported); resistance/strength training; stretching		5 weeks; 3 sessions per week, 50 min per session							Improvement in QoL; ROM Reduction in fatigue; pain
Ho et al 2016 China [32]		> 18 years 0–IV		Total: 104 Received RT: 104		HRQoL/QoL; Self-esteem; mood state; physical functioning; physical symptoms; disease and treatment coping		Questionnaire (self-reported)		Dance		3 weeks, 6 sessions per week, 90 min per session							Improvement in QoL; physical symptoms; mood state; self-esteem
Ho et al 2018 China [33]		Mean: 49.4 years 0–III		Total: 121 Received RT: 121		Fatigue; sleep quality; cortisol levels; pain		Perceived stress scale; BFI; Brief Pain Inventory; PSQI; saliva samples		Dance movement therapy		3 weeks, 2 sessions per week, 90 min per session							Improvement in sleep quality Reduction in fatigue; stress Steeper cortisol slope in dance movement therapy group
Kulkarni et al 2013 [34]		30–60 years I–II		Total: 54 Received RT: 54		HRQoL/QoL; fatigue		BFI; WHOQOL-BREF		Walking; aerobic/cardiovascular (intensity not reported); stretching		6 weeks; 5 sessions per week; 30–50 min per session							Improvement in QoL Reduction in fatigue
Oliveira et al 2009 Brazil [35]		52.7 ± 10.2 years (study), 48.0 ± 10.1 years (control) 0–IV		Total: 69 Received RT: 69		Shoulder/arm ROM; arm circumference; scar tissue formation (adhesion); functional capacity of shoulder		Goniometer; physical therapy protocol; Wingate Scale		Physical therapy—kinesiotherapy		5–6 weeks, 3 sessions per week, 45 min per session							Improvement in ROM; scar tissue No change in arm circumference; functional capacity of shoulder
Mock et a**l** 2001 USA [﻿^36^]		28–75 years IA–IIIA		Total: 50 Received RT: 31		HRQoL/QoL; fatigue; distress; mood state; physical functioning; social functioning		PFS; POMS; MOS-Emotional; MOS-Social; MOS SF-36; MOS-Physical; Walk Test and Activity Level Rating Scale		Walking		6 weeks (during RT or 6 months (during CT), 5–6 sessions per week, 15–30 min per session							Improvement in QoL; physical functioning Reduction in fatigue; stress;
Mock et al 2005 USA [37]		18–70 years 0–III		Total: 119 Received RT: 69		Fatigue; Physical functioning		PFS; MOS SF-36		Walking		6 weeks (during RT) or 3–6 months (during CT), 5–6 sessions per week, 15–30 min per session							Reduction in fatigue
Pickett et al 2002 USA [38]		34–75 years IA–IIIA		Total: 52 Received RT: 33		Fatigue		Investigator-developed diary recorded fatigue level ratings		Walking		6 weeks, 5–6 sessions per week, Started at 10–15 min—advanced to 30 min per session							Not clear
Raghavendra et al 2009 India [39]		30–70 years II–III		Total: 88 Received RT: 88		Anxiety; depression; stress; cortisol levels		Saliva sample; HADS; perceived stress scale		Yoga		6 weeks, 3 sessions per week, 60 min per session							Reduction in anxiety; depression; stress; cortisol levels
Torres et al 2023 Brazil [40]		> 18 years 0–III		Total: 156 Received RT: 156		Fatigue		FACIT-F		Mat Pilates		3–6 weeks (alongside RT), 2 sessions per week, 60 min per session							Reduction in pain No change in fatigue
Vadiraja et al 2009 India [41]		30–70 years I–III		Total: 88 Received RT: 88		HRQoL/QoL		EORTC QLQ C-30; PANAS		Yoga		6 weeks, minimum 3 sessions per week, 60 min per session							Improvement in QoL
VanderWalde et al 2021 USA [42]		> 65 years 0–III		Total: 54 Received RT: 54		Fatigue; physical functioning		FSI, PROMIS, SPPB		Walking		4–6 weeks (alongside RT), 3–5 sessions per week, 10–30 min per session							Reduction in fatigue
Wilkie et al 2022 USA [43]		> 18 years 0–IV		Total: 109 for BC Received RT: 134 (NR how many were BC patients)		Fatigue		Schwartz Cancer Fatigue Scale		Self-reported varied exercise		4 weeks, sessions varied per participant, 15–30 min per session							No change in fatigue
Ratcliff et al 2016 USA [44]		> 18 years 0–III		Total: 163 Received RT: 163		HRQoL/QoL; insomnia/trouble sleeping; depression; cortisol rhythmicity; post-traumatic responses		SF-36; CES-D Scale; Impact of Event Scale; saliva samples		Yoga		6 weeks, 3 sessions per week, 60 min per session							Improvement in QoL; depression; sleep quality
Yang et al 2015 Taiwan [45]		Mean: 50.3 years NR		Total: 58 Received RT: 58		Fatigue		BFI		Aerobic/cardiovascular (low intensity); Aerobic/cardiovascular (moderate intensity)		6 weeks, 3 sessions per week, 50–60 min per session							Reduction in fatigue
Zhang et al 2023 China [46]		> 18 years I–IV		Total: 184 Received RT: 184		HRQoL/QoL; fatigue; anxiety		HADS; EORTC QLQ C-30; CFS		Aerobic/cardiovascular (intensity not reported); resistance/strength training; stretching		5 weeks, 7 sessions per week, 60 min per session							Improvement in QoL Reduction in fatigue; depression No change in anxiety
**Continued after cessation of active RT treatment/not specified**																			
Anderson et al 2012 USA [47]		> 18 years I–III		Total: 104 Received RT: 67		HRQoL/QoL; physical functioning; shoulder/arm ROM		PFS; callipers		Walking; aerobic/cardiovascular (intensity not reported); resistance/strength training; stretching		18 months, 2 sessions per week for first 3 months, followed by option to transition to home-based exercise 1 session per week, 65 min per session							Improvement in physical functioning No change to QoL; arm circumference
Battaglini et al 2008 USA [48]		35–70 years ‘Recent Diagnosis’		NR		Fatigue; body composition; total caloric intake		Food dairy; callipers; PFS		Resistance/strength training; stretching; cardiovascular—intensity based on Exercise and Cancer Recovery Guidelines		6 months, 2 sessions per week; 60 min per session							Reduction in fatigue Increase in total caloric intake Change in body composition
Cadmus et al 2009 USA [49]		35–75 years 0–III		Total: Group 1 (IMPACT): 75 Received RT: 16		HRQoL/QoL; anxiety; depression; stress; self-esteem; physical functioning		FACT-B; 2-item Fordyce Happiness Measure; Rosenberg Self-Esteem Scale; CES-D; STAI; Perceived Stress Scale; SF-36		Walking; aerobic/cardiovascular (moderate intensity)		Both groups: 6 months, 2–5 sessions per week, 30 min per session							IMPACT: No change in QoL YES: No change in QoL
				Group 2 (YES): 50 Received RT: 24															
Campbell et al 2005 UK [50]		Average: 47–48 years NR		Total: 16 Received RT: NR		HRQoL/QoL; fatigue; physical functioning		FACT-G; FACT-B; PFS; SWLS		Walking; aerobic/cardiovascular (low intensity); resistance/strength training		12 weeks, 2 sessions per week, ~ 30 min per session							Improvement in QoL; physical functioning Reduction in fatigue
Cešeiko et al 2020 Latvia [51]		18–63 years I–III		Total: 55 Received RT: 49		HRQoL/QoL; physical functioning		The 6-min walking distance; 30 s sit to stand test; Skin-fold calliper		Walking; other: maximal strength training		12 weeks, 2 sessions per week, 20 min per session							Not clear
Cešeiko et al 2019 Latvia [52]		18–63 years I–III		Total: 55 Received RT: 49		HRQoL/QoL		EORTC QLQ C-30; EORTC QLQ-BR23		Resistance/strength training		12 weeks, 2 sessions per week, 20 min per session							Improvement in QoL; fatigue
Emslie et al 2007 UK [53]		40–76 years ‘Early stage’		Total: 203 Received RT: 33		HRQoL/QoL; body image; mood state; physical functioning; shoulder/arm ROM		Focus groups		NR		12 weeks, 2 sessions per week, 30 min per session							Respondents described the benefits they had gained: positive mental states; better physical functioning; improved sleep; improved confidence; feeling more in control
Gollhofer et al 2015 Germany [54]		> 18 years 0–III		Total: 117 Received RT: 117		Fatigue		FAQ		Resistance/strength training		12 weeks, sessions per week, and session durations NR							No change in fatigue
Grabenbauer et al 2016 Germany [55]		> 18 years NR		NR		HRQoL/QoL; body composition		EORTC QLQ C-30; BIA		Aerobic/cardiovascular (intensity not reported)		12 months, 2–3 sessions per week, 30–60 min per session							Improvement in QoL; body composition
Haines et al 2010 Australia [56]		55.9 ± 10.5 years (study), 54 ± 11.5 years (control) NR		Total: 89 Received RT: 82		HRQoL/QoL; fatigue; body composition; upper limb swelling		EORTC QLQ C-30; Multi-dimensional Fatigue Index; BIA		Aerobic/cardiovascular (intensity not reported); resistance/strength training; shoulder mobility and balance		Weeks/sessions per week NR -36 min DVD instruction + 20 min of walking							Improvement in QoL; physical functioning Reduction in fatigue; upper limb swelling
Jain et al 2023 India [57]		Mean: 43 years (study) and 47 years (control) II–III		Total: 96 Received RT: 67		Fatigue; physical functioning		EORTC QLQ C-30; EORTC QLQ-FA12		Yoga		48 weeks, 5 sessions per week, 50 min per session							Improvement in fatigue; physical functioning
Kilbreath et al 2012 Australia [58]		Mean: 51.6–53.5 years 0–III		Total: 160 Received RT: 157		Shoulder/arm ROM		Digital inclinometer		Resistance/strength training; stretching		8 weeks, 7 sessions per week, session duration NR							Improvement in ROM
Lee et al 2008 USA [59]		> 18 years I–III		Total: 112 Received RT: 59		Nausea		Self-report diary/log; INVR		Aerobic/cardiovascular (moderate intensity)		12 months, 3 sessions per week, 20 min per session							Reduction in nausea
Lötzke et al 2016 Germany [60]		NR I–III		Total: 92 Received RT: 26		HRQoL/QoL; fatigue; life satisfaction, mindfulness, and spirituality		EORTC QLQ C-30; BLMSS; CFS-D; FMI		Yoga; physical exercise		Both groups: 12 weeks; 3 sessions per week; 20–60 min per session							Improvement in QoL for both groups No change in fatigue; life satisfaction; spirituality and mindfulness for either group
Malik et al 2023 India [61]		32–70 years II–III		Total: 100 Received RT: 100		HRQoL/QoL		QOL-CSV		Walking; aerobic/cardiovascular (moderate intensity)		Weeks/sessions per week NR, 20 min per day							Improvement in QoL (specifically fatigue; pain; sleep quality; constipation)
Mavropalias et al 2023 Australia [62]		32–78 years I–III		Total: 106 Received RT: 106		HRQoL/QoL; fatigue		FACT-B; FACIT-F; PSQI		Aerobic/cardiovascular (moderate intensity); resistance/strength training		12 weeks, 3–5 sessions per week, 20–30 min per session							Improvement in QoL Reduction in fatigue
Rao et al 2017 India [63]		30–70 years II–III		Total: 98 Received RT: 67		HRQoL/QoL; anxiety; depression; physical functioning		STAI; BDI; FLIC		Yoga		24 weeks, 3 sessions per week (during RT), 60 min per session							Improvement in QoL Reduction in anxiety; depression; stress; treatment-related symptoms and toxicity
Rao et al 2015 India [64]		30–70 years II–III		Total: 98 Received RT: 67		Depression		BDI		Yoga		24 weeks, 3 sessions per week (during RT), 60 min per session							Reduction in depression
Rao et al 2009 India [65]		30–70 years II–III		Total: 98 Received RT: 67		Anxiety; Symptom distress		STAI; subjective symptom checklist (for treatment-related side effects, problems with sexuality and image, and relevant psychological and somatic symptoms related to breast cancer)		Yoga		24 weeks, 3 sessions per week (during RT), 60 min per session							Reduction in anxiety; symptom distress
Reis et al 2013 USA [66]		> 18 years I–III		Total: 41 Received RT: 41		HRQoL/QoL; fatigue; shoulder/arm ROM		FACT-G, FACIT-F; Goniometer		Aerobic/cardiovascular (intensity not reported); Nia exercise		12 weeks, 3 sessions per week, 20–60 min per session							Reduction in fatigue No change in QoL; ROM
Schmidt et al 2016 Germany [67]		> 18 years 0–III		Total: 160 Received RT: 160		Fatigue; depression; pain		FAQ		Resistance/strength training		12 weeks, 2 sessions per week, 60 min per session							Reduction in fatigue; pain
Spence et al 2022 Australia [68]		> 18 years II–IV		Total: 60 Received RT: 45		HRQoL/QoL		PROMIS		Walking; aerobic/cardiovascular (low intensity); resistance/strength training		12 weeks, 3–4 sessions per week on average, 20–40 min per session							Improvement in QoL
Steindorf et al 2017 Germany [69]		> 18 years 0–III		Total: 160 Received RT: 160		Sleep quality		EORTC QLQ C-30; FAQ		Resistance/strength training		12 weeks, 2 sessions per week, 60 min per session							Improvement in sleep quality
Steindorf et al 2014 Germany [70]		> 18 years 0–III		Total: 160 Received RT: 160		HRQoL/QoL; fatigue; depression		EORTC QLQ C-30; EORTC QLQ-BR23; CES-D Scale		Resistance/strength training		12 weeks, 2 sessions per week, 60 min per session							Improvement in QoL Reduction in fatigue
Vehmanen et al 2022 Finland, Portugal, Israel, and Italy [71]		40–70 years IA–IIIB		Total: 311 Received RT: 247		HRQoL/QoL; physical functioning		HADS; EORTC QLQ C-30; EORTC QLQ-BR23		Aerobic/cardiovascular (low intensity); Aerobic/cardiovascular (moderate intensity)		Varied per participant – self-reported							Improvement in QoL Reduction in depression; anxiety
Winters-Stone et al 2018 USA [72]		> 18 years 0–IV		Total: 90 Received RT: 43		Fatigue; Depression; Mood state		Brief Profile of Mood States		Yoga		8 weeks, 3 sessions per week, 30 min per session							Improvement in mood state; depression Reduction in fatigue
Wiskemann et al 2017 Germany [73]		Mean: 55.2 0–III		Total: 146 Received RT: 146		Fatigue; Weight change; Body composition; Physical functioning; Shoulder/arm ROM		Scales/measurement; FAQ; BMI		Resistance/strength training		12 weeks, sessions NR on							Improvement in ROM Reduction in weight (slight) No change in fatigue
‘**Nutrition**-**only’ studies**																			
Klement et al 2020 Germany [74]		18–75 years NR		Total: 63 Received RT: 63		HRQoL/QoL; Weight change; Body composition		Self-report diary/log; BIA; Blood sample; EORTC QLQ C-30; EORTC QLQ-BR23						Ketogenic diet		Consultation and education (handouts) at baseline only		Generic (all participants receive same advice)	Improvement in QoL Decreased body weight
Rockenbach et al 2011 Brazil [75]		35–77 years 0–III		Total: 40 Received RT: 24		Weight change; Oxidative stress; BMI		Scales/measurement; blood sample; FFQ adapted from the Sichieri and Everhart validated questionnaire						Guidance on healthy eating; changes in dietary intake pre and post treatment; weight/body composition		Consultation at baseline only		Generic (all participants receive the same advice)	Dietary intake changes (increase in meat and eggs, dairy products, beans, oils, and fats) Increased body weight Increase in stress
**Combined exercise and nutrition studies**																			
**During active RT treatment only (period in which patient is receiving RT)**																			
Klement et al 2021 Germany [76]	< 75 years NR		Total: 44 Received RT: 44		HRQoL/QoL; weight change; body composition; other: vitamin D levels, metabolic blood parameters and hormones		Self-report diary/log; BIA; blood sample; EORTC QLQ C-30; Diary log kept for both nutrition and exercise		Walking; Bike rides		‘Length of RT’ – weeks NR, 7 sessions per week, 30 min per session		Palaeolithic diet		Education (handouts) at baseline only		Generic (all participants receive same advice)		Improvement in QoL Increase in vitamin D Decreased body weight Reduction in free T3 hormone levels; blood glucose; triglycerides; low-density lipoprotein cholesterol; C-reactive protein levels
**Continued after cessation of active RT treatment**																			
Carayol et al 2019 France [77]	18–75 years I–III		Total: 143 Received RT: 143		HRQoL/QoL; fatigue; anxiety; depression; body composition; BMI		BIA; GPAQ; Device (accelerometer); HADS; EORTC QLQ C-30; MFI; Nutritional Analysis Software; 10-point visual analogue scale		Resistance/strength training; stretching; cardiovascular—intensity based on Exercise and Cancer Recovery Guidelines		6 months, 2 sessions per week; 60 min per session		Healthy eating based on World Cancer Research Fund recommendation		Application NR—baseline and periodic		Generic (all participants receive same advice)		Improvement in QoL; depression; anxiety Reduction in fatigue; BMI
Jacot et al 2020 France [78]	> 18 years 0–IV		Total: 360 Received RT: 360		HRQoL/QoL; Fatigue; Weight change; Body composition		Self-report diary/log; GPAQ; Nutritional Analysis Software; HADS; EORTC QLQ C-30, MFI; MFI-20		Walking; Jogging/running; aerobic/cardiovascular (moderate intensity); resistance/strength training; dancing; stretching; cycling; swimming		26 weeks (during CT and RT), 2 sessions per week, 60 min per session		Healthy eating based on World Cancer Research Fund recommendation		6 consultations (during CT and RT), each consultation involved an evaluation of nutritional status, nutrition care tailored to the patient		Tailored – consultations taught principles of a well-balanced diet, fostered weight control during treatment and induced appropriate feeding behaviours after treatment		No change in QoL; fatigue; depression; anxiety; body weight Dietary intake changes (increase in fibre and reduction in animal proteins and alcohol)
Kirkham et al 2019 Canada [79]	29–77 years 0–III		Total: 73 Received RT: 67		HRQoL/QoL; weight change; body composition		Scales/measurement; FACT-B; SF-36		Aerobic/cardiovascular (intensity not reported); resistance/strength training		Phase 1: Length of CT/RT, 3 sessions per week, 15–30 min per session		Achieving adherence to Canada’s Food Guide and Canadian Cancer Society guidelines, and Dietary Reference Intake		Consultation (with dietitian) at baseline only—2-h session		Generic (all participants receive same advice)		Improvement in QoL No change in body weight
											Phase 2: 10 weeks, 2 sessions per week, 15–30 min per session								
											Phase 3: 10 weeks, 1 session per week, 15–30 min per session								

*BDI* Beck’s Depression Inventory, *BFS* Benefit Finding Scale, *BIA* bioelectrical impedance analysis, *BMI* body mass index, *BC* breast cancer, *BFI* Brief Fatigue Inventory, *BMLSS* Brief Multidimensional Life Satisfaction Scale, *CFS-D* Cancer Fatigue Scale, *CFS* Cancer-related Fatigue Scale, *CES-D* Centre for Epidemiologic Studies Depression Scale, *CT* chemotherapy, *EORTC QLQ FA12* EORTC Core QoL Cancer Related Fatigue Questionnaire, *EORTC QLQ C-30* EORTC Core QoL Questionnaire, *EORTC QLQ-BR23* European Organisation for Research and Treatment of Cancer-Breast Module, *FAQ* Fatigue Assessment Questionnaire, *FSI* Fatigue Symptom Inventory, *FMI* Freiburg Mindfulness Inventory, *FACT-B* Functional Assessment of Cancer Therapy-Breast, *FACT-G* Functional Assessment of Cancer Therapy-General, *FACIT-F* Functional Assessment of Chronic Illness Therapy-Fatigue questionnaire, *FLIC* Functional Living Index of Cancer, *GPAQ* Global Physical Activity Questionnaire, *HADS* Hospital anxiety and depression scale, *IES* Impact of Events Scale, *INVR* Index of Nausea, Vomiting, and Retching, *MOS SF-36* Medical Outcomes Study Short Health Form, *MFI* Multidimensional Fatigue Inventory, *NR* not reported, *PROMIS* Patient Reported Outcomes Measurement Information System, *PFS* Piper Fatigue Scale, *PSQI* Pittsburgh Sleep Quality Index, *PANAS* Positive and Negative Affect Schedule, *POMS* Profile of Mood States, *QOL-CSV* Quality of Life Patient/Cancer Survivor Version

### Settings and geographical locations

Of the 81% (*n* = 47) of studies that reported on intervention settings, 24% (*n* = 14) were carried out in the hospital where the participant was receiving radiation therapy, 31% (*n* = 18) were undertaken at home, and 14% (*n* = 8) begun in a hospital/supervised setting before being completed at home. Other settings included clinics or study centres (10%; *n* = 6) and gymnasiums or recreation centres (10%; *n* = 6). Most studies were performed in the United States of America (29%; *n* = 17) or Germany (15%; *n* = 9).

### Participant characteristics

Collectively, a total of 5352 women with breast cancer were included, of which 4615 received radiation therapy (some studies only had a portion of participants receiving radiation therapy). Ages ranged between 18 and 78 years. Breast cancer diagnoses included in most studies (74%; n = 43) were stage I–III; the remaining studies either included up to stage IV or did not report this information. In addition to radiation therapy, participants had surgery (79%; *n* = 46 studies), chemotherapy (83%; *n* = 48 studies), and/or hormone therapy (22%; *n* = 13 studies), with 93% (*n* = 54) of studies reporting participants received two or more treatment types. Only 36% (*n* = 21) of studies reported the radiation therapy treatment technique and/or prescription participants received, with the majority prescribed 50 Gy/25# (either three-dimensional conformal radiation therapy or intensity-modulated radiation therapy) and a boost.

### Quality of life measures

For all 58 studies, the main outcomes measured were general quality of life (57%; *n* = 33), fatigue (52%; *n* = 30), and depression (24%; *n* = 14). For these, 24% (*n* = 14) used the European Organisation of Research and Treatment of Cancer (EORTC) Core QoL Questionnaire (EORTC QLQ C-30), 28% (*n* = 16) used the Brief Fatigue Inventory (BFI), and 10% (*n* = 6) used the Centres for Epidemiological Studies Depression Measure (CES-D). Some studies measured anthropological aspects via callipers, devices (such as armbands), digital inclinometers, and saliva and blood samples. All outcome measures and results are reported in Table 2.

### Exercise-only studies

Of the 58 studies, most interventions (90%; *n* = 52) were ‘exercise-only’ (Table 2). The most common exercise interventions were aerobic/cardiovascular training with intensities ranging from low to moderate (28%; *n* = 16 of the 58 total), resistance/strength training (24%; *n* = 14), yoga (22%; *n* = 13), and walking (21%; *n* = 12). Less-utilised exercise interventions included stretching, dancing/dance movement, Mat Pilates, Kinesiotherapy, Qigong, and Nia exercise.

Many exercise-only interventions were implemented over 6 weeks (28%; *n* = 16) precisely corresponding with the participant’s radiation therapy treatment regimen/prescription. Furthermore, 47% (*n* = 27) of exercise-only studies interventions either continued past cessation of active RT treatment or did not specify the length of intervention (Table 2 divides studies based on intervention length). Exercise frequency ranged from 2 to 7 times per week and session duration ranged from 20 to 90 min. For 22% (*n* = 13) of the studies, a qualified instructor supervised the exercise sessions, 21% (*n* = 12) were supervised by a physiotherapist or exercise physiologist, and 9% (*n* = 5) were supervised by a registered nurse.

**Fig. 2:**
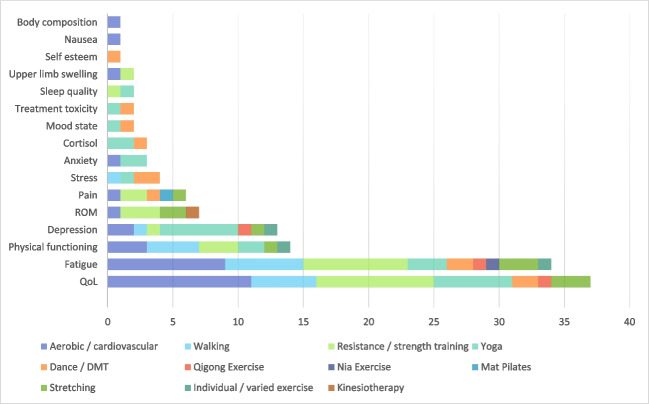
Relationship between exercise type and outcome, for ‘exercise-only’ studies

Studies that implemented interventions with aerobic/cardiovascular exercise and resistance/strength training were those to find the most improvements in QoL (11 of 16 studies and 9 of 14, respectively). This was also found in the improvement of fatigue (9 of 16 studies that implemented aerobic exercise and 8 of 14 for resistance/strength). It is noteworthy that the studies that implemented yoga resulted in a wide range of psychological improvements, such as depression, stress, anxiety, sleep quality, and cortisol levels, in addition to QoL and fatigue. Studies that utilised interventions such as Kinesiotherapy and Mat Pilates only found improvement in shoulder ROM and pain, respectively. All relationships between exercise interventions and outcomes can be seen in Fig. 2.

### Nutrition-only studies

Only two studies in this review were ‘nutrition-only’ interventions. Klement et al. implemented a Ketogenic (or Keto) diet which consisted of giving all participants generic advice to adhere to the Keto diet. This study found decreased body weight and improved QoL. The remaining study, by Rockenbach et al., comprised participants receiving guidance on healthy eating (following the Dietary Guide for the Brazilian Population) at baseline only. Increased body weight and increased stress were reported in this study.

### Combined exercise and nutrition studies

Four studies implemented exercise and nutrition as combined interventions. Two studies, by Carayol et al. and Jacot et al., comprised education and counselling to meet the World Cancer Research Fund [80] recommendations, in conjunction with planned supervised aerobic/cardiovascular and muscle strength exercise, throughout the duration of the participants’ treatment. While these studies implemented the same intervention, the outcome measures were different. One study found improvement in QoL and reduction in fatigue; the other found no change in QoL, fatigue, depression, anxiety, and body weight. Kirkham et al. implemented a singular 2-hour, group-based education session on healthy eating, to align with Canada’s Food Guide, Canadian Cancer Society Guidelines, and Dietary Reference Intake. The exercise component consisted of supervised aerobic and whole-body resistance sessions, with encouragement of home-based exercise to work toward achieving the National Comprehensive Cancer Network recommendations [81]. This study found improvement in QoL and no body weight changes. The final study by Klement et al. recommended the Palaeolithic diet (which consists of the consumption of fatty meats and organ meats from humanely raised animals, wild-caught fish, eggs, nuts and seeds, algae, spices, vegetables, and fruits. Excluding processed foods, grains of all types, legumes, and vegetable oils except for native coconut and olive oil and dairy products except for ghee) in conjunction with outdoor activity (walking or bike riding), 30 min per day. It was found that this intervention increased vitamin D and improved QoL. In addition, reduced body weight, T3 hormone levels, blood glucose, triglycerides, low-density lipoprotein cholesterol, and C-reactive protein levels were reported.

## Discussion

This scoping review aimed to find literature reporting on exercise and/or nutritional interventions implemented during radiation therapy for women with breast cancer. It was discovered that most studies utilised exercise interventions (90%; *n* = 52) while only a small proportion had nutrition interventions (3%; *n* = 2). Moreover, only a limited number of studies (7%; *n* = 4) have explored the potential benefits of combining these interventions. While exercise and nutrition interventions are extensively recognised in cancer prevention and survivorship settings [82, 83], this review demonstrates that they are not routinely used during treatment and rarely together.

### Active treatment vs. survivorship

In the systematic review by Barchitta et al., combined exercise and nutritional interventions are investigated in breast cancer survivors (post-active treatment) for the effect on QoL outcomes [84]. It was reported that most studies recommended various combinations of regular daily activity and healthy diets. More specifically, multiple studies utilised the American Cancer Society dietary guidelines (to consume vegetables, fruits, and whole grains regularly) [85], while other studies provided dietary counselling or recommendations [86]. Alongside this, several studies implemented supervised aerobic exercise regimes [87, 88], while others gave exercise or physical activity advice [89]. These studies consistently reported positive outcomes, such as reduced fatigue and depression, improved QoL, and enhanced overall emotional functioning in survivors [84].

However, comparing the QoL of survivors and those actively receiving radiation therapy can be difficult. This is due to the acute side effects of treatment and the potential effect on the QoL of patients during the active treatment stage. A two-phase study by Van Leeuwen et al. reported findings from a review of literature surrounding cancer survivorship and responses to interviews of disease-free cancer survivors. It was reported that most acute disease symptoms and treatment-related side effects had resolved by approximately 12 months following treatment. These findings led to the development of a questionnaire specifically designed to target survivors, excluding questions typically intended to capture acute disease and treatment-related complications, as they were considered irrelevant to survivors [90]. Similarly, a qualitative study by Bloom et al. reports on interviews with 185 women with breast cancer (55%; *n* = 101 received RT) at two different timeframes: during initial diagnosis/treatment and then 5 years later. Findings indicate that participants’ physical and mental well-being improved between the different stages, especially in the areas of surgical side effects, body image concern, and ‘worry about the future’ [91]. Ensuring comprehensive support is accessible to patients during survivorship is crucial and a necessary focus, due to the increasing survival rate of breast cancer. However, interventions such as exercise and nutrition that have shown benefits during active treatment could be implemented much earlier, engaging patients during their treatment and aiming to continue into survivorship.

### Exercise and nutrition in cancer

A systematic review by Baguley et al. found 20 studies addressing the effect of nutrition therapy and exercise on cancer-related fatigue and QoL in men with prostate cancer during treatments. Consistent with our review, most studies focussed on exercise-only interventions (80%; *n* = 16), and the remaining 20% were either nutrition-only (5%; *n* = 1) or a combination of both (15%; *n* = 3). Of the exercise-only interventions, they found aerobic, or resistance training programs were the most utilised [92]. Likewise, in our review, aerobic training (20%; *n* = 10) and resistance training (22%; *n* = 11) were the most reported. While this review closely reflects the results presented in our review, it is important to note that the needs of women with breast cancer and men with prostate cancer can be vastly different. Baguley et al. focused on measures of cancer-related fatigue and overall quality of life, whereas our review aimed to capture all aspects of QoL possible. This approach was taken to provide comprehensive evidence to optimally support women holistically. A review by Browall et al. reported on 17 qualitative studies focusing on exercise during or after chemotherapy treatment for breast cancer. The interventions were group-based or personalised programs and comprised mindfulness movement, aerobics, and resistance training. Browall et al. found decreased fatigue, stress, and increased confidence and sense of control were common outcomes among the studies reviewed [93]. Similarly, a systematic review by Gilmour et al. reported on nutritional support for women receiving chemotherapy for breast cancer. They identified several chemotherapy-induced nutritional challenges women face, including changes in taste, appetite, nutritional status, and weight gain. Approaches to supporting women by providing nutritional interventions and education were also reviewed, affirming these practices may influence eating habits and improve treatment side effects [94].

### Implementing exercise and nutritional support

The nutrition-focused studies outlined in this scoping review illustrated changes in eating patterns (e.g., Palaeolithic diet, Ketogenic diet) implemented during RT treatment. However, as there were no measures of habitual dietary intake at baseline, the impact of these changes is difficult to interpret. In addition, most were administered with minimal or no support from qualified dietitians. As women with breast cancer receiving radiation therapy are in vulnerable circumstances, this level of change to a person’s eating patterns could be contra-indicated and lead to outcomes that are not conducive to the treatment phase, rather than an improvement. Not having the appropriate qualified professionals supporting the changes could also impose another level of stress. A qualitative study by Landmark et al. describes the experiences of ten women with newly diagnosed breast cancer, regarding social support. It was reported that participants found the ‘informative’ dimension (information, advice, and counselling) of social support instrumental in their ability to manage the burden of living with breast cancer [95]. Furthermore, Keaver et al. report a mixed methods (survey and focus groups) study regarding nutritional support and intervention preferences of cancer survivors, for which 27% (*n* = 15) of participants professed to be interested in receiving nutritional advice via live online sessions, followed by 20% (*n* = 11) preferring face-to-face personalised advice [96]. Therefore, we can see there is reported interest in nutrition to support the treatment journey in breast cancer; however, what types of changes are best suited to women with breast cancer receiving radiation therapy are not clear.

## Implications for future research

The findings of this scoping review provide valuable insights for informing practice, by mapping the current literature regarding exercise and/or nutrition interventions to improve QoL for women with breast cancer receiving RT. The primary focus of our review is shown to be on the impact of exercise on general QoL, fatigue, and depression. However, it is important to acknowledge that nutritional input and interventions also play a crucial role in ensuring the well-being of women with breast cancer, and this is a gap in the literature found by this review. Furthermore, there is a growing body of evidence that exercise and nutrition can decrease the chance of recurrence and improve cancer outcomes. In the context of women with breast cancer undergoing RT treatment, future research could explore the potential benefits of supporting women with exercise and nutrition intervention/s, as a standard approach, to not only enhance their QoL but improve their outcome.

## Conclusion

In conclusion, this review highlights the existing literature on investigations into the relationship between exercise, nutrition, and the QoL of women with breast cancer receiving RT, this review underscores that the existing evidence is not comprehensive, and further research in this area is warranted to improve women’s QoL and overall health and well-being.

## Strengths and limitations

The study selection process can cause bias, due to the subjective judgement of reviewers.

## Supplementary Information

Below is the link to the electronic supplementary material.

Supplementary file1 (DOCX 22 KB)

## Data Availability

No datasets were generated or analysed during the current study.
