# Supplementary material for: The effectiveness of exercise and/or nutritional interventions to improve the quality of life of women with breast cancer receiving radiation therapy: a scoping review
Source: Support Care Cancer. 2024 Oct 23;32(11):745. doi: 10.1007/s00520-024-08933-1 (PMC11499338; doi:10.1007/s00520-024-08933-1)
Supplement: Supplementary file 1 — Supplementary file1 (DOCX 22 KB) [file 520_2024_8933_MOESM1_ESM.docx]

**Supplementary Table 1. Full Search Strategy**

| Medline | |
| --- | --- |
| 1 | breast cancer.mp. or Breast Neoplasms/ |
| 2 | Carcinoma, Ductal, Breast/ |
| 3 | radiation therapy.mp. or Radiotherapy/ |
| 4 | Radiation Oncology/ |
| 5 | breast radiotherapy.mp. |
| 6 | Diet/ or "Diet, Food, and Nutrition"/ |
| 7 | nutrition.mp. |
| 8 | food.mp. |
| 9 | exercise/ or muscle strength/ or physical endurance/ or physical fitness/ or "range of motion, articular"/ |
| 10 | physical activit*.mp. |
| 11 | dance therapy/ or exercise therapy/ or endurance training/ or muscle stretching exercises/ or resistance training/ |
| 12 | relaxation therapy/ or yoga/ |
| 13 | dancing/ or sports/ |
| 14 | Qigong/ |
| 15 | Exercise/ or exercise.mp. |
| 16 | Tai Ji/ |
| 17 | flexibility.mp. or Pliability/ |
| 18 | case-control studies/ or controlled before-after studies/ |
| 19 | clinical study/ or clinical trial/ or controlled clinical trial/ or randomized controlled trial/ or comparative study/ |
| 20 | Randomized Controlled Trial/ |
| 21 | controlled clinical trial/ |
| 22 | 1 or 2 |
| 23 | 3 or 4 or 5 |
| 24 | 6 or 7 or 8 or 9 or 10 or 11 or 12 or 13 or 14 or 15 or 16 or 17 |
| 25 | 18 or 19 or 20 or 21 |
| 26 | 22 and 23 and 24 and 25 |

| Embase | |
| --- | --- |
| 1 | breast cancer/ or breast tumor/ or breast carcinoma/ |
| 2 | breast cancer.mp. |
| 3 | radiotherapy/ |
| 4 | cancer radiotherapy/ |
| 5 | radiation oncology/ |
| 6 | breast radiotherapy/ |
| 7 | diet/ or nutrition/ |
| 8 | food/ |
| 9 | exercise/ or aerobic exercise/ |
| 10 | circuit training/ or endurance training/ or exercise intensity/ or muscle exercise/ or pilates/ or resistance training/ |
| 11 | "Range of motion"/ |
| 12 | fitness/ |
| 13 | physical activity/ |
| 14 | dance therapy/ or dancing/ |
| 15 | yoga/ |
| 16 | qigong/ |
| 17 | Tai Chi/ |
| 18 | pilates/ |
| 19 | randomized controlled trial/ or controlled clinical trial/ |
| 20 | case control study/ or clinical study/ or controlled study/ |
| 21 | clinical trial/ or clinical study/ |
| 22 | 1 or 2 |
| 23 | 3 or 4 or 5 or 6 |
| 24 | 7 or 8 or 9 or 10 or 11 or 12 or 13 or 14 or 15 or 16 or 17 or 18 |
| 25 | 19 or 20 or 21 |
| 26 | 22 and 23 and 24 and 25 |
| CINAHL | |
| S1 | breast cancer |
| S2 | breast cancer or breast neoplasm or breast carcinoma or breast tumor |
| S3 | breast lesion |
| S4 | radiation therapy or radiotherapy |
| S5 | radiation, therapy: breast cancer |
| S6 | breast radiotherapy |
| S7 | breast radiation therapy |
| S8 | diet and nutrition |
| S9 | food |
| S10 | nutrition or diet or food or nourishment or food intake or eating |
| S11 | exercise or physical activity or fitness |
| S12 | physical activity or exercise or fitness or physical exercise |
| S13 | range of motion or rom or range of movement or flexibility |
| S14 | dance movement therapy or dmt or dance or movement |
| S15 | endurance training |
| S16 | resistance training or strength training or weight training or resistance exercise |
| S17 | yoga or yoga therapy or yoga exercise |
| S18 | pilates or pilates exercise or pilates training |
| S19 | qigong |
| S20 | running or jogging or run or jog |
| S21 | (walking group or walking program ) OR walking |
| S22 | tai chi or tai chi chuan or tai ji quan |
| S23 | flexibility or range of motion |
| S24 | randomized controlled trials or rtc or randomised control trials |
| S25 | quasi-experimental design or experimental or randomized, controlled trial. |
| S26 | case control study or case-control study |
| S27 | pre and post intervention |
| S28 | clinical trial |
| S29 | S1 OR S2 OR S3 |
| S30 | S4 OR S5 OR S6 OR S7 |
| S31 | S8 OR S9 OR S10 OR S11 OR S12 OR S13 OR S14 OR S15 OR S16 OR S17 OR S18 OR S19 OR S20 OR S21 OR S22 OR S23 |
| S32 | S24 OR S25 OR S26 OR S27 OR S28 |
| S33 | S29 AND S30 AND S31 AND S32 |
| Scopus | |
| ( ( breast AND cancer ) OR ( breast AND carcinoma ) OR ( breast AND neoplasms ) ) AND ( ( radiation AND therapy ) OR ( radiotherapy ) OR ( radiation AND oncology ) OR ( breast AND radiotherapy ) ) AND ( ( exercise ) OR ( physical AND activity ) OR ( resistance AND training ) OR ( yoga ) OR ( pilates ) OR ( qigong ) OR ( dance ) OR ( muscle AND strengthening ) OR ( physical AND fitness ) OR ( range AND of AND motion ) OR ( relaxation ) OR ( tai AND chi ) OR ( walking ) OR ( running ) OR ( diet ) OR ( nutrition ) OR ( food ) ) AND PUBYEAR > 2000 AND LANGUAGE ( english ) AND TITLE-ABS-KEY-AUTH ( randomised AND controlled AND trials OR quasi-randomised AND controlled AND trials OR case AND controls ) | |
| Cochrane | |
| 1 | Breast Neoplasms |
| 2 | Carcinoma, ductal, breast |
| 3 | Radiotherapy |
| 4 | Diet |
| 5 | Diet, Food and Nutrition |
| 6 | Exercise |
| 7 | Resistance Training |
| 8 | Qigong |
| 9 | Tai Ji |
| 10 | Pliability |
| 11 | Range of Motion, Articular |
| 12 | Case Control Studies |
| 13 | Clinical Trial |
| 14 | Randomised Clinical Trials |
| 15 | Comparative Studies |
| 16 | Clinical Studies |
| 17 | #1 OR #2 OR #3 |
| 18 | #4 OR #5 OR #6 OR #7 OR #8 OR #9 OR #10 OR #11 |
| 19 | #12 OR #13 OR #14 OR #15 OR #16 |
| 20 | #17 AND #18 AND #19 |
